# Heck’s Disease in Saudi Patients: A Case Series and Literature Review

**DOI:** 10.7759/cureus.98825

**Published:** 2025-12-09

**Authors:** Waad Bin Khanin, Areen AlMardawi, Reaam AlZeer, Asmaa Faden

**Affiliations:** 1 College of Dentistry, King Saud University, Riyadh, SAU; 2 Oral Medicine and Diagnostic Science, College of Dentistry, King Saud University, Riyadh, SAU

**Keywords:** case report, focal epithelial hyperplasia, heck’s disease, hpv infection, saudi female

## Abstract

Heck’s disease, also known as focal epithelial hyperplasia, is a rare benign oral mucosal condition primarily associated with human papillomavirus (HPV) infection. It typically presents as single or multiple, soft, well-delimited sessile papules or nodules that share the same color as the surrounding mucosa. The condition is more frequently observed in females and predominantly affects children. Although it was initially identified among Native American, Inuit, and African populations, Heck’s disease is now recognized worldwide. Some studies have suggested a possible genetic predisposition linked to the human leukocyte antigen (HLA)-DR locus.

This report describes three cases of Heck’s disease. The first case is a 70-year-old female patient who presented with four whitish, smooth, round lesions located on the upper and lower labial mucosa and the left lateral surface of the tongue. The second case concerns a 39-year-old female patient who exhibited a single white, slightly raised, dome-shaped nodule with a smooth surface on the right side of her tongue. The third case describes a 49-year-old female patient who presented with a pinkish-white lesion measuring approximately 0.3 mm on the left lateral border of the tongue. All three cases were confirmed histopathologically as Heck’s disease following excisional biopsy. Notably, two of the patients were sisters, suggesting the potential role of familial or genetic factors in the disease’s pathogenesis.

In conclusion, this case series contributes to the limited literature on Heck’s disease and underscores the need for further research into its genetic associations, particularly among the Saudi population. Comparing the clinical and genetic characteristics of these cases with previously documented cases worldwide may provide valuable insight into the disease’s etiology and distribution patterns.

## Introduction

Heck’s disease, also known as multifocal or focal epithelial hyperplasia, is a rare, benign oral condition caused primarily by human papilloma virus (HPV) types 13 and 32. It presents as single or multiple soft, well-delimited sessile papules or nodules of the same color as the surrounding oral mucosa (i.e., white to pinkish). The nodule surfaces may be smooth or irregular and nonkeratinized. Lesion diameters range from 1 to 10 mm, and the lesions may coalesce to form large cobblestone-like areas. They typically disappear when the mucosa is stretched and reappear when the tension is released [1-3]. Commonly affecting the labial and buccal mucosa, tongue, and gingiva (rarely the palate and tonsillar region), the disease is observed more frequently in females and predominantly impacts children, due to their underdeveloped immune systems [3].

Although usually self-limiting, Heck’s disease persists in some cases, potentially affecting quality of life. The lesions tend to be fewer and flatter in adults than in children. Historically, the condition was found primarily in Native American, Inuit, and African populations, but it is now recognized globally, with some evidence suggesting a genetic predisposition linked to the human leukocyte antigen (HLA)-DR locus [3]. Heck’s disease occurs more commonly in South and North America and some Asian regions than in Europe and Africa [3,4]. Despite its global distribution, there remains a clear knowledge gap in the Gulf Region, where published cases are limited and diagnostic approaches such as routine molecular testing are not consistently applied. Therefore, documenting such cases contributes valuable information to the epidemiology and clinical understanding of this condition.

## Case presentation

Case 1

A 70-year-old Saudi woman presented to the Oral Medicine Clinic at a dental university hospital in Riyadh with the chief complaint of a lesion on the inner surface of her upper lip, which appeared 10 months ago. She reported that the lesion was bothersome and that she occasionally bit on it. The patient had histories of diabetes mellitus, hypertension, and dyslipidemia.

**Figure 1:**
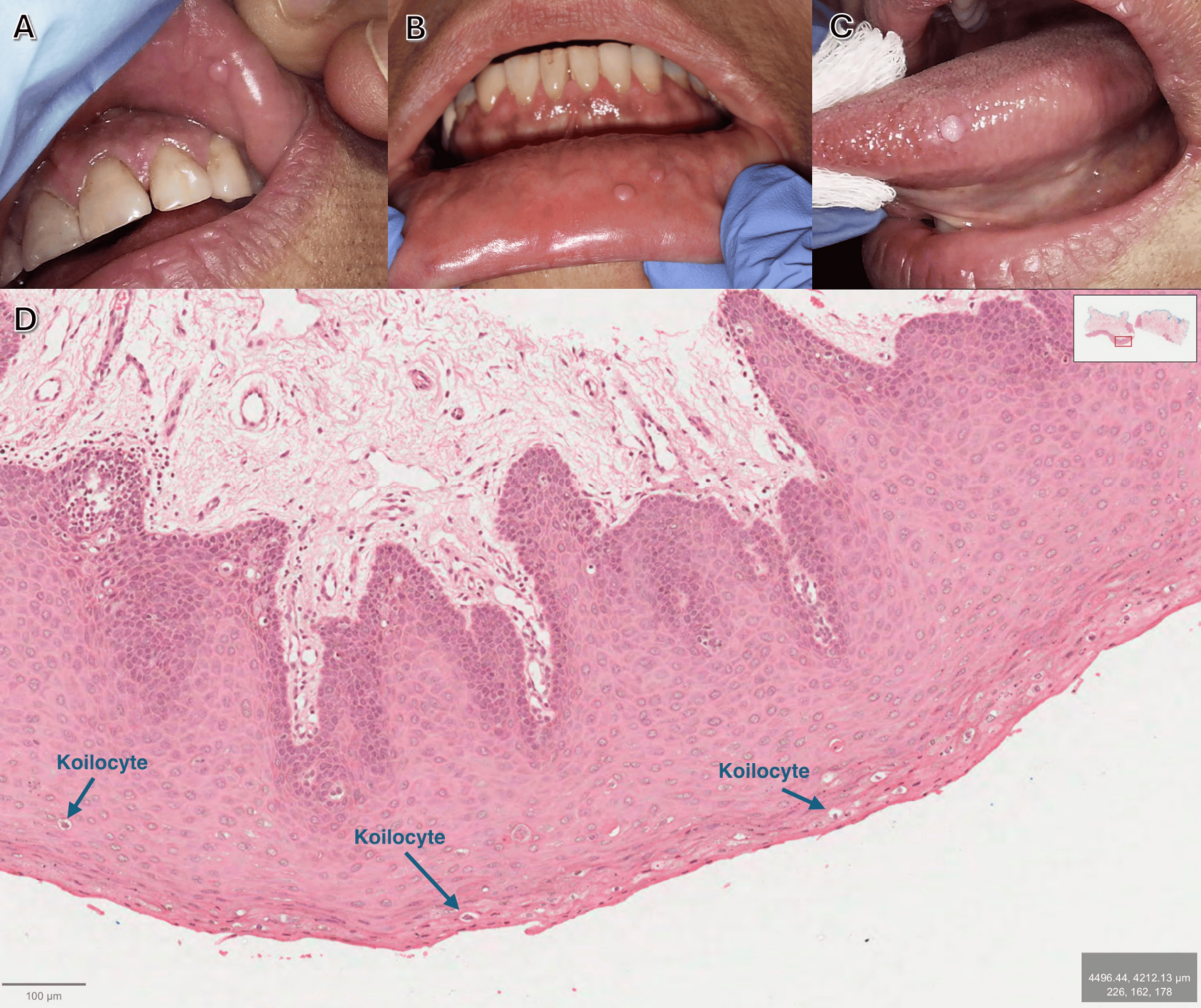
Case 1 H&E: hematoxylin and eosin (A) Clinical photograph showing a whitish, smooth, round nodule on the upper labial mucosa measuring approximately 5 mm in greatest diameter based on direct clinical measurement with a graduated periodontal probe. (B) Two whitish, smooth nodules on the lower labial mucosa, each measuring approximately 5 mm, measured clinically with a graduated periodontal probe. (C) Well-defined, smooth, whitish nodule on the left lateral aspect of the tongue measuring approximately 5 mm, measured clinically with a graduated periodontal probe. (D) Medium-power H&E-stained section (×10) showing acanthosis, hyperkeratosis, and elongated hyperplastic rete ridges. Labeled blue arrows indicate koilocytes within the stratified squamous epithelium. The underlying stroma shows fibrous connective tissue with vascular proliferation and focal hemorrhage

Intraoral examination revealed a total of four lesions: one nodule on the upper labial mucosa, two nodules on the lower labial mucosa, and one nodule on the left lateral surface of the tongue (Figure 1). The whitish, smooth, round lesions measured approximately 5 mm in greatest diameter, based on clinical measurement with a graduated periodontal probe prior to excision. They were painless, with no associated symptoms. No cervical lymphadenopathy was detected.

Based on the clinical presentation, the differential diagnosis for this case included Heck’s disease (focal epithelial hyperplasia) and multiple fibromas. All of the lesions were surgically excised under local anesthesia because of patient-reported discomfort, repeated traumatic irritation, and the need for definitive histopathological diagnosis; the specimens were placed in formalin and submitted for histopathological analysis. No HPV typing was attempted (Figure 1). It revealed a nodular configuration with stratified squamous epithelial surfaces exhibiting hyperkeratosis, acanthosis, and edematous changes. Basal cell melanosis and melanin incontinence were present. The rete ridges were hyperplastic, broad, and elongated, surrounding cores of connective tissue. Scattered koilocytes and a few mitosoid cells were observed in the basaloid squamous epithelium. A separate tiny nodular proliferation displayed similar epithelial features. The underlying stroma demonstrated hyperplastic, dense, fibrous connective tissue with spindle-shaped fibroblasts, blood vessels of various calibers, and areas of profuse hemorrhage. Deeper sections of the specimen revealed skeletal muscle fibers and neurovascular bundles. These features confirmed the diagnosis of Heck’s disease.

Case 2

A 39-year-old Saudi woman presented to the Oral Medicine Clinic with the chief complaint of pain on both sides of the tongue, especially the left side, when eating. The patient had noticed a lesion on the left side of her tongue one year ago, but had no systemic symptoms and had not undergone previous treatment. She had asthma and used an albuterol inhaler; she had no other relevant medical condition.

**Figure 2:**
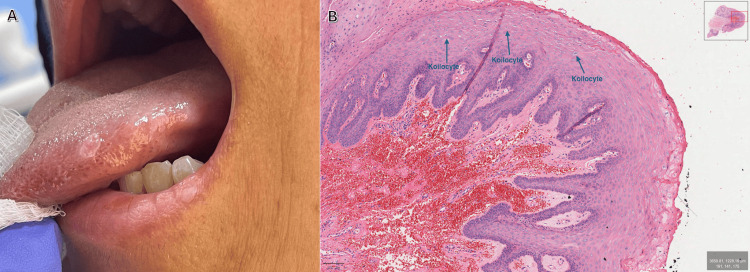
Case 2 H&E: hematoxylin and eosin (A) Solitary, well-circumscribed white, dome-shaped nodule on the left side of the tongue measuring approximately 3 mm in greatest diameter based on direct clinical measurement with a graduated periodontal probe. (B) Medium-power H&E-stained section (×10) showing acanthosis, hyperkeratosis, and elongated rete ridges. Labeled blue arrows indicate koilocytes. The stroma shows fibrous hyperplasia with increased vascularity and focal hemorrhage

Intraoral examination revealed a solitary, well-circumscribed lesion of approximately 3 mm in greatest diameter, measured clinically with a graduated periodontal probe. (Figure 2). The lesion presented as a white, slightly raised, dome-shaped nodule with a smooth surface. The overlying mucosa was intact, with no evidence of ulceration, erythema, or induration. Surrounding tissues, including the adjacent tongue mucosa and dentition, appeared to be within normal limits. An excisional biopsy was performed under local anesthesia because of the patient’s symptomatic presentation and to establish a definitive diagnosis; the specimen was fixed in formalin and submitted for histopathological analysis. HPV typing was not performed. The histopathological analysis revealed the same features as in case 1 (Figure 2), confirming the diagnosis of Heck’s disease.

Case 3

A 49-year-old Saudi woman presented to the Oral Medicine Clinic with a chief complaint of a tongue lesion that had been noticed one year earlier. She had a seven-year history of Crohn’s disease and a three-year history of arthritis. This patient was the biological sister of the patient described here as case 2.

**Figure 3:**
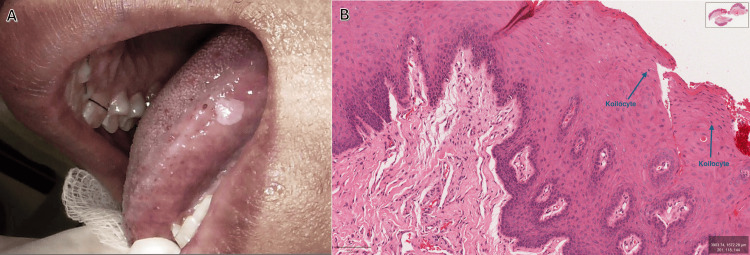
Case No. 3. H&E: hematoxylin and eosin (A) Well-circumscribed pinkish-white lesion with a soft texture on the left lateral border of the tongue measuring approximately 3 mm in greatest diameter, measured clinically with a graduated periodontal probe. (B) Medium-power H&E-stained section (×10) showing mild hyperparakeratosis, acanthosis, and broad, confluent rete ridges. Labeled blue arrows indicate koilocytes with perinuclear clearing, balloon cells, and mitosoid cells. The stroma shows reduced fibrous tissue, variable vascularity, and deeper muscle, nerve, and adipose tissue

Intraoral examination revealed a painless, pinkish-white lesion measuring approximately 3 mm in greatest diameter on the left lateral border of the tongue, based on direct clinical measurement using a graduated periodontal probe (Figure 3). The lesion had a soft consistency.

An excisional biopsy was performed under local anesthesia to establish a definitive histopathological diagnosis and exclude other clinically similar lesions, and the specimen was fixed in formalin for analysis; HPV typing was not performed. Histopathological analysis of the excised tissue revealed that the mucosa was covered by stratified squamous epithelium that demonstrated mild hyperparakeratosis and prominent acanthosis (Figure 3). The rete ridges were broad and elongated; the lesional rete pegs were at the same depth as the adjacent normal pegs and had a club-shaped and confluent appearance. High-power magnification revealed koilocytosis; the keratinocytes had pyknotic nuclei and were surrounded by clear areas, balloon cells, and a few mitosoid cells. The subjacent lamina propria showed a loss of fibrous connective tissue and variable vascularity. Skeletal muscle, nerve fibers, and adipose tissue were observed at the base of the specimen. These features confirmed the diagnosis of multifocal epithelial hyperplasia (Heck’s disease).

## Discussion

Heck’s disease prevalence rates vary significantly among regions and populations. In South America, early reported rates were 7.4% among Brazilian indigenous tribes [3] and up to 21% among the Waimiri Atroari Indians [3]. In other parts of the world, varying rates of 19.4% for the indigenous Nanortalik population in Greenland [3], 1.6% in Mexican mestizo populations [3], and 0.1% in southern Mexico’s Maya region [3] have been reported. Prevalence rates are lower in Europe, with the rate of 0.11% reported for the general Caucasian population of Sweden [3]. The rate of 8.6% was reported for Canada [3], and the reported prevalence for Argentina is 0.02%, reaching 0.09% in some provinces [3].

Heck’s disease is uncommon in the Middle East, and few cases have been documented in Saudi Arabia and the Gulf region (Table 1). One case was documented in a 21-year-old woman from Al-Hofuf, Saudi Arabia, in 2020 [5]. The patient presented with asymptomatic papules on her lower lip, which had gradually increased in size over two years [5]. Histopathological examination confirmed the diagnosis of focal epithelial hyperplasia, revealing typical koilocytic changes, and dermoscopy revealed polymorphic vascular patterns [5]. The patient subsequently initiated cryotherapy for cosmetic purposes [5]. In 2012, a 43-year-old Saudi woman was diagnosed with multifocal epithelial hyperplasia (Heck’s disease) after presenting with cobblestone-like papules on her lips and buccal mucosa [6]. Histopathological examination confirmed the diagnosis, and immunohistochemical analysis revealed positivity for HPV and Ki-67 markers [6]. The patient was referred for further evaluation of her gastrointestinal symptoms [6]. Another case of Heck’s disease was documented in a seven-year-old girl in Saudi Arabia in 2007 [7]. The patient was otherwise in good health, with no underlying medical condition, and her family history was insignificant [7]. In 1978, a case of focal epithelial hyperplasia (Heck’s disease) in an 11-year-old girl in Abu Dhabi was reported [8]. This case had a familial background and was the first documented occurrence in the United Arab Emirates [8].

**Table 1:** Summary of previously reported Saudi and regional cases of Heck’s disease compared with the present case series NR: not reported; IHC: immunohistochemistry The table outlines patient age, sex, lesion site, comorbidities, family relations, histopathologic features, and management

Reference	Age	Sex	Lesion site	Comorbidities	Family relations	Histologic features	Management
(Saudi Arabia, 2020) [5]	21 yrs	Female	Lower lip	None	NR	Koilocytic changes consistent with focal epithelial hyperplasia	Cryotherapy for cosmetic purposes
(Saudi Arabia, 2012) [6]	43 yrs	Female	Lips and buccal mucosa	Gastrointestinal symptoms	NR	Features of multifocal epithelial hyperplasia; HPV and Ki-67 positive on IHC	Observation and referred for further GI evaluation
(Saudi Arabia, 2007) [7]	7 yrs	Female	Oral mucosa	None	Insignificant family history	NR	NR
(Abu Dhabi, 1978) [8]	11 yrs	Female	NR	NR	Positive familial background	NR	NR
Case no. 1	70 yrs	Female	One nodule on the upper labial mucosa, two nodules on the lower labial mucosa, and one nodule on the left lateral surface of the tongue	Diabetes mellitus, hypertension, and dyslipidemia	Insignificant family history	Acanthosis, hyperkeratosis, elongated hyperplastic rete ridges, koilocytes within the stratified squamous epithelium	Excisional biopsy
Case no. 2	39 yrs	Female	Left side of the tongue	Asthma	Positive familial background	Acanthosis, hyperkeratosis, elongated hyperplastic rete ridges, koilocytes within the stratified squamous epithelium	Excisional biopsy
Case no. 3	49 yrs	Female	Left side of the tongue	Crohn’s disease and arthritis	Positive familial background	mild hyperparakeratosis, acanthosis, broad confluent rete ridges, koilocytes with perinuclear clearing, balloon cells, and mitosoid cells	Excisional biopsy

HPV subtypes have been identified as etiological agents for Heck’s disease, but factors governing disease susceptibility remain incompletely understood. A genetic tendency for increased susceptibility to infection with HPV subtypes 13 and 32 has been hypothesized [9]. Recent studies have revealed a significant association between focal epithelial hyperplasia and HLA-DR4 (DRB1*0404) [10,11]. This allele, which frequently occurs in Native American descendants, was linked to Heck’s disease in a Mexican population [1], and its frequency was reported to be >80% in another population with the disease [3]. As a major histocompatibility complex II molecule, it may be incapable of binding to certain HPV-13 and HPV-32 viral proteins, increasing the susceptibility to clinical infection by these subtypes and thus the risk of focal epithelial hyperplasia development [10,11].

Horizontal transmission via infected food and eating utensils, associated with poor hygiene in groups with low socioeconomic status, has also been reported. [12] This route may clarify the tendency of HPV-13 and HPV-32 to colonize the oral cavity, which is uncommon for HPV subtypes except those that are sexually transmitted (HPV-6, HPV-11, and HPV-16) [12]. These considerations lead to chronic immunodeficiency, which allows the existence and transfer [13].

The differential diagnosis for Heck’s disease may encompass condyloma acuminatum, inflammatory fibrous hyperplasia, juvenile papillomatosis, neurofibromatosis, tuberous sclerosis, and syndromes, including Gorlin-Goltz syndrome, focal dermal hyperplasia syndrome, and multiple endocrine neoplasia (MEN) [2].

The clinical presentation together with histopathological findings can be relied on for the diagnosis of Heck’s disease [1,14], but biopsy remains the gold standard for final diagnosis [3,15]. Cytopathic changes (particularly koilocyte-like cells and nuclear inclusions) support an HPV-related etiology. Additional investigations include molecular techniques such as general-primer polymerase chain reaction (PCR), type-specific PCR, direct DNA sequencing, type-specific in-situ DNA hybridization, and PCR-based DNA chip assay [2].

The treatment of Heck’s disease is individualized according to the patient’s age, sex, esthetic needs, and immune status; the disease severity; the buoyancy of the patient and/or caregiver; operator expertise; and the modalities available. Options include conservative observation, pharmacological therapy, conventional surgical excision with or without adjuvant energy sources, and chemical cauterization [2]. Conservative treatment options for Heck’s disease that have yielded promising results involve oral tretinoin therapy [16], vitamin A supplementation [17], the use of topical keratolytic agents [18], interferon-β administration [18], and the topical administration of the immune response modifier imiquimod [19]. Interferon-α has also been used for topical, intralesional, and systemic treatment, with varying degrees of success [20]. Trichloroacetic acid is one of the most effective agents for noninvasive treatment to resolve Heck’s disease lesions [21].

Persistent or recurrent lesions may be treated with chemical cautery, surgery, or medical approaches. When surgery is indicated (e.g., for esthetically unpleasing non-resolving lesions), scalpel excision is effective but is associated with bleeding, pain, edema, and medication needs. Laser surgery (CO2 [22,23] and diode [4] laser excision) offers hemostasis, reduced postoperative discomfort and edema, and surface disinfection and typically obviates sutures and scar formation; it thus achieves patient satisfaction. Other reported options include electrocauterization (successful), cryotherapy (mixed results), and radiotherapy, which is generally avoided due to the risks of anaplastic cellular changes and malignant transformation [2,22,24,25].

This report brings the total number of documented cases of Heck’s disease in Saudi patients to six (Table 1). All of these cases occurred in female patients [5-7], suggesting that the disease shows female predominance in the Saudi population, as reported for other ethnic groups [3]. Two of the cases reported here occurred in sisters, suggesting the involvement of familial factors. Familial clustering may be linked to household behaviors such as the sharing of food, utensils, and toothbrushes, which facilitates the salivary transmission of HPV-13 [3]. The typical clinical presentation of Heck’s disease was observed in all three cases reported here, supporting the internal validity of the diagnoses. All lesions in these three cases were managed by scalpel excision, as this option was the most suitable for the patients’ situations.

This case report, together with previously reported cases, highlights opportunities for future research on related genetic factors, including Saudi ethnicity, and the exploration of whether the observed patterns resemble those reported for Heck’s disease in other populations.

## Conclusions

This case series describes Heck’s disease in three Saudi patients, with diagnoses confirmed by histopathological analysis after excisional biopsy. Two of the patients were sisters, which may suggest a possible familial predisposition; however, no molecular or HLA data are available to support a definitive genetic implication. The disease remains rare in the Arab population, and environmental factors may also play a role. Although this condition is benign, its unusual appearance can cause understandable concern for patients and their families. These cases emphasize the importance of maintaining clinical awareness and confirming the diagnosis by biopsy to avoid misdiagnosis and unnecessary interventions. Additionally, Heck’s disease is generally self-limiting and often resolves without the need for treatment unless lesions are symptomatic or esthetically concerning. Despite this, recurrence has been reported, which highlights the value of patient education and appropriate follow-up. Future studies incorporating molecular analysis, targeted genetic testing, and HPV typing are needed to better clarify the disease’s pathogenesis and improve patient care.
